# HTLV-1 in rural Guinea-Bissau: prevalence, incidence and a continued association with HIV between 1990 and 2007

**DOI:** 10.1186/1742-4690-7-50

**Published:** 2010-06-04

**Authors:** Carla van Tienen, Maarten F Schim van der Loeff, Ingrid Peterson, Matthew Cotten, Birgitta Holmgren, Sören Andersson, Tim Vincent, Ramu Sarge-Njie, Sarah Rowland-Jones, Assan Jaye, Peter Aaby, Hilton Whittle

**Affiliations:** 1 Medical Research Council, Fajara, The Gambia; 2 Municipal Health Service and Academic Medical Centre, Amsterdam, The Netherlands; 3 Department of Laboratory Medicine, Division of Medical Microbiology/Virology, Lund University, Lund, Sweden; 4 Swedish Institute of Infectious Disease Control, Stockholm, Sweden; 5 Weatherall Institute of Molecular Medicine, Human Immunology Unit, John Radcliffe Hospital, Oxford, UK; 6 Projecto de Saúde de Bandim, Indepth Network, Bissau, Guinea-Bissau

## Abstract

**Background:**

HTLV-1 is endemic in Guinea-Bissau, and the highest prevalence in the adult population (5.2%) was observed in a rural area, Caió, in 1990. HIV-1 and HIV-2 are both prevalent in this area as well. Cross-sectional associations have been reported for HTLV-1 with HIV infection, but the trends in prevalence of HTLV-1 and HIV associations are largely unknown, especially in Sub Saharan Africa. In the current study, data from three cross-sectional community surveys performed in 1990, 1997 and 2007, were used to assess changes in HTLV-1 prevalence, incidence and its associations with HIV-1 and HIV-2 and potential risk factors.

**Results:**

HTLV-1 prevalence was 5.2% in 1990, 5.9% in 1997 and 4.6% in 2007. Prevalence was higher among women than men in all 3 surveys and increased with age. The Odds Ratio (OR) of being infected with HTLV-1 was significantly higher for HIV positive subjects in all surveys after adjustment for potential confounding factors. The risk of HTLV-1 infection was higher in subjects with an HTLV-1 positive mother versus an uninfected mother (OR 4.6, CI 2.6-8.0). The HTLV-1 incidence was stable between 1990-1997 (Incidence Rate (IR) 1.8/1,000 pyo) and 1997-2007 (IR 1.6/1,000 pyo) (Incidence Rate Ratio (IRR) 0.9, CI 0.4-1.7). The incidence of HTLV-1 among HIV-positive individuals was higher compared to HIV negative individuals (IRR 2.5, CI 1.0-6.2), while the HIV incidence did not differ by HTLV-1 status (IRR 1.2, CI 0.5-2.7).

**Conclusions:**

To our knowledge, this is the largest community based study that has reported on HTLV-1 prevalence and associations with HIV. HTLV-1 is endemic in this rural community in West Africa with a stable incidence and a high prevalence. The prevalence increases with age and is higher in women than men. HTLV-1 infection is associated with HIV infection, and longitudinal data indicate HIV infection may be a risk factor for acquiring HTLV-1, but not vice versa. Mother to child transmission is likely to contribute to the epidemic.

## Background

Human T-cell Lymphotropic Virus type 1 (HTLV-1) is the first retrovirus linked to human disease and was isolated for the first time in 1979 [1]. HTLV-1 is an ancient infection and certain subtypes may have been present in humans for > 5,300 years [2]. Although HTLV-1 has spread worldwide, it is only endemic in distinct regions including south-western Japan, the Caribbean and countries from Sub-Saharan Africa [3]. HTLV-1 causes adult T-cell leukemia (ATL) and tropical spastic paresis (also called HTLV-1 associated myelopathy) (TSP/HAM) in up to 5% of infected individuals which can lead to prolonged morbidity (TSP/HAM) and death (ATL). HTLV-1 is also associated with other inflammatory syndromes such as uveitis and infective dermatitis [4,5]. Infectious complications such as tuberculosis have also been reported to be higher in HTLV-1 infected compared to uninfected individuals [6,7]. Although the majority of infected individuals are lifelong asymptomatic carriers, increased mortality has been observed in HTLV-1 infected individuals compared to non-infected individuals in community studies [8-12].

The routes of transmission of HTLV-1 are: mother-to-child transmission (especially prolonged breast feeding), sexual intercourse, blood transfusion and sharing of needles and syringes [13]. HTLV-1 prevalence typically increases with age and is higher in women than men, a pattern that is similar to that of HIV-2 but different from HIV-1. It remains unclear what effect HTLV-1 has on disease progression in HIV-1 and HIV-2 [14,15]. Despite the shared routes of transmission, the prevalence of HTLV-1 may decrease while that of HIV-1 increases within the same population [16,17]. Studies have shown HIV-1 prevalence is increasing and HIV-2 prevalence is decreasing in many countries in West Africa [18-24], but trends in HTLV-1 prevalence are largely unknown.

HTLV-1 is endemic in Guinea-Bissau, and the highest prevalence in the adult population was 5.2% in 1990 in Caió, the rural area studied in this paper [25]. The trends of HTLV-1 prevalence and incidence, factors associated with HTLV-1 infection, and associations with HIV-1 and HIV-2 in this area between 1990 and 2007 were determined.

## Results

### Participation in the three surveys

In the 2007 survey, 2895 people participated out of the 3907 adults that were registered in the census [26]. This coverage of 74.1% was similar to previous surveys: 2770 of 3775 (73.4%) registered adults participated in 1990 [27] and 3110 out of 4127 registered adults (75.4%) participated in 1997. In all three surveys more women participated than men, which reflects the imbalance in the men-women ratio in the census registrations, caused by men migrating out of the area [27,28]. In 1997, 49% of non-participants were women compared to 61% of participants (p < 0.001); the median age of non-participants (30 years) was lower than that of participants (33 years; p = 0.002). In 2007, 54% of non-participants were women compared to 60% of participants (p = 0.001), while the median age did not differ between the groups (both 31 years; p = 0.23).The main reason for non-participation in all surveys was short-term travel (< 6 months). Refusal to give a blood sample was the second cause for non-participation, and this declined from 8.7% in 1997 to 5.0% (p < 0.001) in 2007 and was similar for both sexes [26].

### HTLV-1 prevalence

The HTLV-1 prevalence was 5.2% (adjusted: 5.5%) in 1990, went up to 5.9% (adjusted: 5.9%) in 1997 (prevalence ratio [PR] 1997 vs. 1990: 1.12, 95% confidence interval [CI] 0.90-1.40) and decreased to 4.6% in 2007 (PR 2007 vs. 1997: 0.78, CI 0.62-0.97). Seven samples in 1997 and 10 samples in 2007 were indeterminate (ELISA positive and PCR negative). The prevalence increased with age for both sexes in all three surveys (score test for trend p < 0.001 for all surveys). Women had a higher prevalence than men in all three surveys (5.8% vs. 4.2% in 1990, p = 0.08; 7.3% vs. 3.6% in 1997, p < 0.001; 5.5% vs. 3.1% in 2007, p = 0.002). The sex-specific prevalence varied by age (test for interaction, p = 0.006 in 1990, p = 0.09 in 1997 and p = 0.004 in 2007).

**Figure 1:**
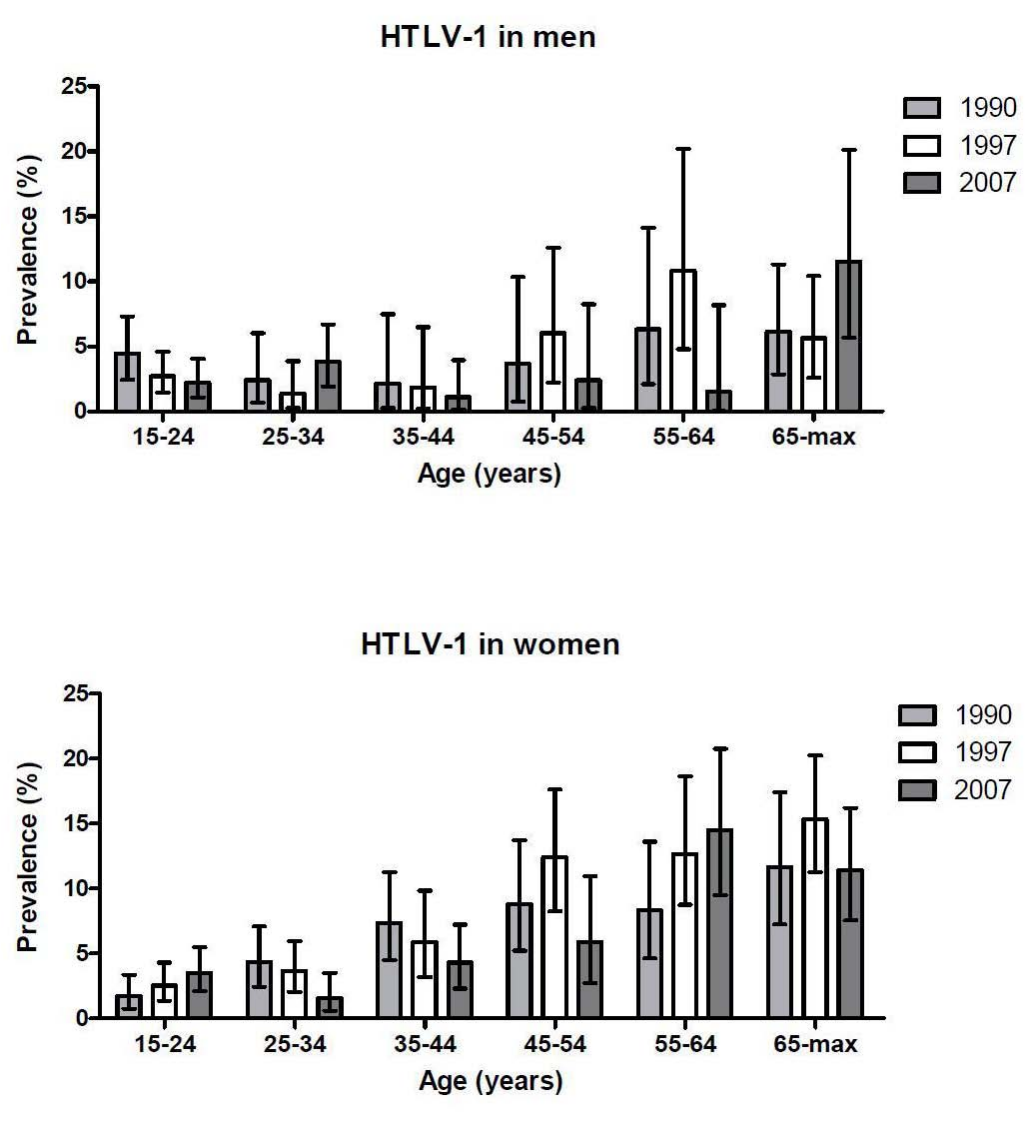
**HTLV-1 prevalence by age and survey year (1990, 1997 and 2007) in men and women in Caió, Guinea-Bissau**. Bars represent 95% confidence intervals; the 1990 figures differ slightly from the figures in B. Holmgren et al., JAIDS 2003, due to use of a different age variable.

In men, the prevalence peaked in the age group 55-64 years in 1990 (6.3%) and 1997 (10.8%) and peaked in the oldest age group (65 + years) in 2007 (11.5%) (Figure 1, Additional file 1). In women, the highest prevalence was observed in the oldest age groups in 1990 (11.6%) and 1997 (15.3%) and in the 55-64 years group in 2007 (14.5%; Figure 1, Additional file 1).

### Factors associated with HTLV-1 infection

The univariate ORs for HTLV-1 infection in 1997 are shown in Additional file 1. For men, in the multivariable model only HIV infection (odds ratio [OR] 2.8, CI 1.3-6.2) and age ≥ 45 years (OR 2.9, CI 1.5-5.6) remained significant. For women, in the multivariable analysis, age ≥ 45 years (OR 2.8, CI 1.8-4.5), HIV infection (OR 4.2, CI 2.7-6.4), being widowed (OR 2.4, CI 1.0-5.6) or divorced (OR 3.1, CI 1.1-9.0) and living in the central area of the village (OR 2.7, CI 1.6-4.3) remained significant.

The univariate ORs for HTLV-1 infection in 2007 are shown in Additional file 1. For men, in the multivariable analysis, TPHA positivity (OR 3.1, CI 1.3-7.2) remained significant and being widowed (OR 6.3, CI 1.3-29.1; p = 0.08) borderline significant; however HIV status (OR 1.2, CI 0.3-4.0; p = 0.8) and older age (OR 1.1, CI 0.4-2.8; p = 0.8) were no longer associated with HTLV-1 infection. For women, in the multivariable analysis only older age (OR 3.3, CI 2.2-5.2) and HIV infection (OR 2.4, CI 1.4-4.1) were significantly associated with HTLV-1 infection.

### HTLV-1 prevalence in mothers and their adult children

HTLV-1 prevalence among study subjects whose mother participated in the same survey round was determined. In 1990, the HTLV-1 prevalence among subjects with a HTLV-positive mother was 9.3% (4/43) versus 3.2% (18/571) among subjects with a HTLV-negative mother (p = 0.04). In 1997, this was 13.7% (14/102) vs. 2.5% (21/832) (p < 0.001) and in 2007 this was 8.3% (5/60) vs. 2.4% (21/876) (p < 0.001). The OR of HTLV-1 infection among subjects with an HTLV-1 infected mother compared to those with an HTLV-1 negative mother was 4.6 (CI 2.6-8.1, p < 0.001). When HIV status was added to this model as a possible confounder, the OR was 4.5 (CI 2.5-7.8). The OR was higher among subjects aged 15-44 (OR 5.5, CI 3.1-9.9) than among subjects older than 44 (OR 1.5, CI 0.3-7.6).

### HTLV-1 incidence

Of the 2501 subjects with a confirmed HTLV status in the 1990 survey, 1306 (52%) provided a blood sample in the 1997 survey. Reasons why a second blood sample was not obtained (n = 1195) were: death (267; 22.3%), migration (479; 40.1%), short-term absence (184; 15.4), refusal (115; 9.6%), insufficient sample (56; 4.7%), no re-identification possible (51; 4.3%) and missing data (43; 3.6%).

Of the 2967 people with a confirmed HTLV status in the 1997 survey, 1308 (44%) provided a sample in 2007. Reasons why a second blood sample was not obtained (n = 1659) were: death (502; 30.3%), migration (690; 41.6%), short-term absence (360; 21.7%), refusal (77; 4.6%), no re-identification possible (26; 1.6%) and missing data (4; 0.2%).

In the first period (1990-1997), 402 men and 833 women were HTLV-1 negative at baseline. Sixteen people became newly infected with HTLV-1 giving an incidence rate (IR) of 1.8/1,000 person-years-of-observation [PYO] (CI 1.1-2.9) (Table 1). In the second period (1997-2007), 436 men and 812 women were HTLV-1 negative at baseline. Eighteen people acquired HTLV-1 infection giving an incidence rate of 1.6/1,000 PYO (CI 1.0-2.5). The IR remained stable (incidence rate ratio [IRR], comparing second vs. the first period, 0.9, CI 0.4-1.7), although there were differences between the sexes and age groups (interaction period and age, p = 0.7; interaction period and sex, p = 0.2).

**Table 1:** HTLV-1 incidence rates per 1000 PYO by sex and age in the periods 1990-1997 and 1997-2007 in Caió, Guinea-Bissau

Sex and age^a^(years)	IR (n/PYO) per 1000 PYO First period 1990-1997	IR (n/PYO) per 1000 PYO Second period 1997-2007	IRR (95% CI) Comparing second to first period
**Men**			

15-44	1.1 (2/1,753)	2.3 (7/3,081)	2.0 (0.4-9.6)
45-max	1.8 (2/1,135)	1.0 (1/968)	0.6 (0.1-6.5)
**Total**	**1.4 (4/2,887)**	**2.0 (8/4,050)**	**1.4 (0.4-4.7)**
**Women**			
15-44	1.5 (6/3,884)	0.4 (2/4,916)	0.3 (0.1-1.3)
45-max	2.7 (6/2,199)	3.0 (8/2,627)	1.1 (0.4-3.2)
**Total**	**2.0 (12/6,083)**	**1.3 (10/7,543)**	**0.7 (0.3-1.6)**
**Men + Women**			
15-44	1.4 (8/5,363)	1.1 (9/7,997)	0.8 (0.3-2.1)
45-max	2.4 (8/3,333)	2.5 (9/3,595)	1.0 (0.4-2.7)
**Overall**	**1.8 (16/8,970)**	**1.6 (18/11,592)**	**0.9 (0.4-1.7)^b^**

PYO, person-years of observation; IR, incidence rate; IRR, incidence rate ratio; CI, confidence interval

^a^age at baseline

^b^adjusted for age and sex by Poisson regression

### HTLV-1 and HIV incidence by retroviral status

In total, data from 835 men and 1512 women were available for analysis (Table 2). Six people acquired HTLV-1 among 260 people that were HIV infected at baseline (IR 4.6, CI 2.1-10.3). Among the 1974 subjects that were HIV negative at baseline, 29 people became HTLV-1 infected (IR 1.6, CI 1.1-2.2). The IRR, adjusted for sex and age, was 2.5 (CI 1.0-6.2) (p = 0.07).

**Table 2:** HTLV-1 incidence by HIV status and HIV incidence by HTLV-1 status in Caió between 1990 and 2007^a^

	IR (Cases/PYO) of HTLV-1 per 1000 PYO	IR (Cases/PYO) of HIV per 1000 PYO^b^	IRR (95% CI) Crude	P value	IRR (95% CI) Adjusted^c^	P value
**HIV status**						
**HIV negative**	1.6 (29/18,607)	-	1		1	
**HIV positive^d^**	4.6 (6/1,303)	-	2.6 (1.1-6.3)	0.03	2.5 (1.0-6.2)	0.07
**HTLV-1 status**						
**HTLV-1 negative**	-	7.7 (141/18,252)	1		1	
**HTLV-1 positive**	-	9.7 (6/621)	1.2 (0.5-2.7)	0.7	1.2 (0.5-2.7)	0.7

PYO person-years of observation; IR Incidence Rate; IRR Incidence Rate Ratio; CI Confidence Interval

^a^data from all subjects that were seen at least twice during the serosurveys and/or case-control studies (see Methods)

^b^subjects that went from single to dually HIV infected were excluded

^c^adjusted for sex and age

^d^includes subjects that are HIV-1, HIV-2 or HIV-1/2 dually positive

Six people became HIV infected among 98 HTLV-1 positive subjects (IR 9.7, CI 4.3-21.5). Among 2047 HTLV-1 negative people, 141 subjects became newly infected with HIV (IR 7.7, CI 6.5-9.1). The IRR, adjusted for sex and age, was 1.2 (CI 0.5-2.7).

## Discussion

### Key findings

This is the largest community based study which has measured the prevalence and incidence of HTLV-1 and its associations with HIV. These data show a high HTLV-1 prevalence of approximately 5% and a stable incidence of approximately 1.7 per 1000 pyo in the adult population of Caió in rural Guinea-Bissau between 1990 and 2007. The prevalence increased with age and was higher in women than in men. A significant association between prevalent HIV and HTLV-1 infections was observed among women, which persisted after adjustment for potentially confounding risk factors. In the longitudinal analysis, HIV positive individuals tended to have a higher risk of acquiring HTLV-1 infection than HIV negative people, while HTLV-1 infection did not increase the risk of becoming infected with HIV.

### HTLV-1 infection

Although the HTLV-1 prevalence in the study area declined from 5.9% in 1997 to 4.6% in 2007, there was little difference between the prevalence in 1990 and 2007. Also, it was still twice as high as the prevalence in the capital Bissau, situated at a distance of 100 km (2.3% in 2006) [16]. Quite big differences in HTLV-1 prevalence have been described between areas that are relatively close geographically, which could be related to cultural and/or ethnic differences [29]. The prevalence is also high compared to other community-based studies in Africa [13]. Studies from Bissau have shown HTLV-1 to be associated with an increased mortality and tuberculosis among HIV infected individuals [11,30] and in the study area with tropical spastic paresis [31]. Hence, it is likely that HTLV-1 has an important impact on this community.

While HTLV-1 and HIV-2 prevalences have decreased, HIV-1 prevalence has increased in both the study area and the capital [16,23,26]. Therefore, it seems unlikely that safer sex practices have played an important role in this decline. An increase in risk behavior and blood transfusions during the War of Independence (1963-74) is thought to have enabled the spread of HIV-2 [32,33]. A concomitant iatrogenic spread through vaccination campaigns and large-scale parenteral treatment programs might have also contributed to the initial spread [34,35]. A similar scenario has been proposed for the spread of hepatitis C virus [36]. These events may also have contributed to the higher prevalence of HTLV-1 observed in earlier studies and the decrease in prevalence observed in the current study.

In this study, sexual risk factors (HIV and TPHA positivity) were mainly identified for women and prevalence was higher among women, which suggests greater susceptibility to HTLV-1 infection by women [37,38]. A positive TPHA test was used as an indication of exposure to syphilis at some point, but does not indicate acute infection.

Mother-to-child-transmission (MTCT) of HTLV-1 has been clearly documented in Japan and Jamaica, but has only been described in one cohort from Africa [39,40]. In the current study, a strong association was observed between HTLV-1 status of mothers and their offspring (OR 4.6, CI 2.6-8.1), indicating that MTCT contributes to maintaining the HTLV-1 epidemic in this community.

Screening of blood transfusions for HIV since 1989 may have lead indirectly to a decrease of HTLV-1 in Bissau [16,41]. This mechanism seems unlikely to have played a role in the Caió area, where having received a blood transfusion was not associated with HTLV-1 infection in either 1990 [25] nor in 1997 (this risk factor was not assessed in 2007).

Striking was the fact that among men, 6 out of the 7 incident cases occurred in 15-16 year olds in 1997-2007. In this area, the incidence of HIV-1 and HIV-2 was low in 15-24 year old men [26], so it remains to be elucidated how these young men acquired HTLV-1.

### HTLV-1 and HIV dual-infection

In this study, HIV and HTLV-1 infections showed a cross-sectional association, as has been shown before in this study area in 1990 [25] and in the general adult population [16,41], elderly people [10], an occupational cohort [42] and pregnant women [43] in Bissau. Why this association is stronger for women than for men remains unclear. Some studies have demonstrated a more efficient male-to-female transmission of HTLV-1 [37,44,45]. An increased susceptibility among older women due to biological changes has been suggested, such as post-menopausal changes in the vaginal mucosa [10,28,38,46]. Higher mortality in men with HIV/HTLV-1 dual infections could also contribute to the observed sex difference; however, mortality rates were similar for men and women in the >35 years cohort from Bissau [11].

It is unknown whether HTLV-1 is a risk factor for HIV infection or vice versa. Therefore, it was interesting to find that pre-existing HTLV-1 infection was not associated with incident HIV infection, but prevalent HIV infection appeared to increase the risk of acquiring HTLV-1. An increased susceptibility could be due to the higher level of immune activation induced by HIV, thereby enhancing the susceptibility of the host to other retroviral infections which are dependent on active immune cells as targets [47-49]. If a substantial number of individuals became HTLV-1 infected perinatally, their HTLV-1 status would not represent a sexual risk factor and this could explain the similar HIV incidence rates observed among HTLV-1 positive and HTLV-1 negative people.

With the current roll-out of anti-retroviral treatment in Guinea-Bissau, it is important to realize that HTLV-1 co-infection may increase the CD4 counts, which is the main indicator for start of treatment [15] (reviewed in [14]).

### Limitations

This study has several limitations. First, in the 1990 and 1997 surveys a number of samples could not be tested for HTLV and these samples were more often of HIV infected people; therefore, the prevalence may have been underestimated. The adjusted prevalences that were reported were based on the assumption that the prevalence of HTLV-1 was distributed the same as among subjects with a known HTLV result. These missing HTLV-1 results may also have led to an underestimation of the association between HTLV-1 and HIV in the reported ORs. Second, the association between HTLV-1 and HIV may have been partly caused by residual confounding, since the factors used in this study may not have controlled completely for (sexual) risk behavior. Third, the HTLV-1 infected adult children of HTLV-1 infected mothers may not have been infected by their mother but might have acquired the virus later in life. However, HIV status was not a confounder in this analysis, suggesting that sexual transmission played a much less important role in this group. Fourth, HIV infected subjects will have had a higher chance of dying before a follow-up blood sample was obtained, especially since the periods between survey rounds were long (median 7.3 years for the first and 9.4 years for the second period). Therefore, the HTLV-1 incidence among HIV positive people is likely to be an underestimate.

Finally, the IRR from the analysis of HIV and HTLV-1 incidence by retroviral status should be interpreted with caution since the number of incident cases among retroviral infected people was small.

## Conclusions

HTLV-1 is highly prevalent in this rural African area and is transmitted both sexually and vertically. Women have a higher prevalence than men and a higher prevalence of HIV/HTLV-1 dual infection. Further studies could help determine whether the association of the two infections is due to behavioral or biological factors. Further studies of HTLV-1 infection in mothers and infants are required for an accurate estimate of the vertical transmission in this area and will help in designing and implementing preventive measures. Public health interventions for safer sex practices need to address all age strata and in particular women who are more at risk for HTLV-1 and HIV infection.

## Methods

### Study population

The study was conducted in the adult population (≥15 years of age) of Caió, a rural area in north-western Guinea-Bissau. Approximately 10,000 people live in Caió, of which approximately 6000 are adults. The village is divided in 10 zones that stretch out over 10 km of cashew forest and rice fields. The main ethnicity (95%) is Manjako and the main belief system is animism with very strong beliefs in ancestor spirits. The population is highly migratory (Bissau, regional in West Africa, Europe). The majority of the population are subsistence farmers engaged in rice, palm oil and cashew nut production. More detailed descriptions have been given previously [26,49].

Demographic surveillance of all residents was initiated in Caió in 1988. The current analyses comprise data of three population surveys among adults carried out in 1989-1991 [25,27], 1996-1998 [49] and 2006-2007 [26]. These surveys are referred to as 1990, 1997 and 2007.

In 1991 a cohort of HIV-positive cases and HIV-negative controls (matched by sex, age and area of living) was initiated; members of this cohort were re-examined in 1996, 2003 and 2006 [15,50-52]. Data from these study rounds in this cohort, together with the results of the population surveys, were used to estimate the HIV incidence by HTLV-1 status and the HTLV-1 incidence by HIV status.

Cohort members have free access to medication and medical care provided by the project's physician who was based permanently at the project. Anti-retroviral treatment for HIV became available in 2007 as part of the national AIDS program, after completion of the 2007 survey.

### Surveys

All adult residents present in Caió during a survey were invited to participate. In short, people were informed about the study, and after they provided consent, they were interviewed and a blood sample was taken. The interview consisted of socio-demographic, sexual behavior and other behavioral questions related to HTLV-1 and HIV. Homes of absent people were re-visited a maximum of two times. Blood samples were transported to Fajara, The Gambia, for HIV, HTLV and syphilis serology testing. The syphilis results were returned to the participants at home and people were invited to visit the project's counselor to obtain their HIV and HTLV results. The 3 surveys have been described in more detail previously [25-27,49]

This study was approved by the Gambia Government/MRC MRC Laboratories Joint Ethics Committee and by the Ministry of Health of Guinea-Bissau.

### Laboratory methods: HTLV testing

For the 1990 survey, two screening ELISAs (Organon Teknika, Boxtel, The Netherlands and Murex I/II, Abbott Murex Diagnostics, Dartford, UK) were used and positive samples were tested with confirmatory assays using PCR and/or Western Blot (Diagnostic Biotechnology HTLV-blot 2.4, Science Park, Singapore) as described [15,25]. Western Blot seropositivity was defined as reactivity against at least two envelope proteins and at least one core protein. For the 1997 and 2007 survey, an ELISA (Murex I/II, Abbott Murex Diagnostics, Dartford, UK) was used to screen all samples. Reactive samples were retested with the same ELISA and were tested with a confirmatory PCR using primer pairs derived from the *tax/rex *gene for the 1997 samples [53] and by nested PCR using primers targeted to either the *gag *p24 open reading frame or to the *tax *gene for the 2007 samples. As a control for DNA quality, all samples testing negative for both p24 and *tax *PCR were shown to be DNA positive using primers against the human beta-2-microglobulin gene (primers listed in Table 3).

**Table 3:** Primers used for HTLV-1 confirmation for the survey in 2007 in Caió, Guinea-Bissau (Methods)

Name	Function	Sequence (5'-3')
mo 076	HTLV-1 p24 OF	TCCCTCCTAGCCAGCCTAC
mo 077	HTLV-1 p24 IF	CATCCAAACCCAAGCCCAGA
mo 078	HTLV-1 p24 IR	CTCCAGTGGCCTGCTTTCC
mo 079	HTLV-1 p24 OR	TCTCGCTTCCAGTGAGTTGG
mo 163	HTLV-1 TAX OF	CGGATACCCAGTCTACGTGTTT
mo 164	HTLV-1 TAX OR	TGAGGGGTTGTCGTCAACGC
mo 165	HTLV-1 TAX IF	CATCTCTGGGGGACTATGTTCG
mo 166	HTLV-1 TAX IR	CTTRACAAACATGGGGAGGAAAT
mo 013	B2M OF	TAGAGGTTCCCAGGCCACTA
mo 014	B2M OR	ACCATGTAGCCTATGCGTGT
mo 015	B2M IF	ACAAGGAGCTCCAGAAGCAA
mo 016	B2M IR	CAGAACATGTCCCCGTCATT

Subjects were considered HTLV-1 positive if at least one of the 2 ELISAs was reactive and either PCR or Western Blot was positive.

### Laboratory methods: HIV testing

In the 1990 survey the HIV diagnoses were determined by serology [27], and most of these samples were confirmed by PCR in a follow-up study in 1991 [51]. For the 1997 survey the following algorithm was used: plasma samples were first screened by ELISA, reactive samples were then tested by ELISA mono-specific for HIV-1 and HIV-2 and by a synthetic peptide-based assay. Dually reactive and indeterminate samples underwent PCR to confirm the HIV status [49]. In the 2007 survey, plasma samples were first screened by ELISA. Subsequently, HIV-1 or HIV-2 confirmation was obtained using a synthetic peptide-based assay. Dually reactive and indeterminate samples were subjected to a different synthetic peptide-based assay. Indeterminate results were resolved using HIV-1 and HIV-2-specific PCR [26].

For all subjects from the three surveys, the HIV status was assessed. In the first survey finger prick blood was collected, so that after HIV testing, samples of several subjects were insufficient for HTLV testing. The proportion without a final HTLV-1 status was higher among the HIV-positive subjects (32%) than among HIV-negative subjects (8%) in the first survey (p < 0.001). Venous blood was collected in 1997, but in some cases these samples were also insufficient for further testing (7% and 4% among HIV-positive and HIV-negative subjects respectively, p = 0.03). In the last survey in 2007, three subjects had a missing HTLV result (all 3 were HIV-negative). Adjusted prevalences for 1990 and 1997 were calculated assuming that the true prevalence of HTLV-1 among HIV infected persons with missing results was the same as among the HIV infected persons with available results (and the same being true for HIV-negative persons).

### Statistical methods

Data were double entered in an Access (Microsoft, Redmond, WA, USA) database and validated. The analysis was performed using Stata 11 (Stata Corporation, College Station TX, USA). The Chi-square test was used to compare proportions. The prevalence ratio (PR) and 95% confidence intervals (CI) were calculated to assess changes in prevalence. Log binomial regression was used to calculate the age- and sex-adjusted PR. Binomial 95% confidence intervals were calculated for the prevalence shown in the figures.

Logistic regression was used to analyze associations between HTLV-1, HIV and other variables. Variables that were associated with HTLV-1 at p ≤ 0.2 in either sex in the univariate analysis, were regarded as potential confounders and were entered into the initial multivariable model. Only general HIV status (so not HIV-1 and HIV-2 separate) was used in the multivariable model. Variables were dropped one-by-one if they were not significantly associated with HTLV-1 and if their omission did not change the Odds Ratio (OR) of the main exposure (HIV) by ≥ 10%. Models were compared with likelihood ratio tests. Age and HIV status were forced into the models.

A general estimation equation model was used to analyze the association of HTLV-1 infection in mother-child pairs, adjusting for clustering (one mother having several children). If children were seen in more than one survey, only the first observation was used in the model.

For the calculation of the incidence rates, it was assumed that HTLV-1 and HIV infections occurred midway between the last seronegative and the first seropositive sample. Incidence rate ratios and 95% CI were calculated using Poisson regression. To estimate the HIV incidence among HTLV-1 positive and HTLV-1 negative people and the HTLV-1 incidence among HIV positive and HIV negative people, the data of subjects were used that were seen at least twice in the surveys or cohort study rounds. Eight subjects acquired HIV and HTLV-1 infection in the same period; because it was unknown which infection came first, these subjects were excluded from the analysis.

Age was either split into 2 or 6 age groups and was treated as a categorical variable.

The STROBE guidelines were followed to report the findings in this article [54].

## Competing interests

The authors declare that they have no competing interests.

## Authors' contributions

SRJ and AJ planned the 2007 survey. CvT carried out the 2007 survey. MSvdL carried out the 1997 survey. MSvdL and IP participated in the data analysis, interpretation and writing of the manuscript. BH initiated the HTLV-1 research in Caió. MC was responsible for HTLV-1 and HIV testing and designed primers for HTLV-1 for the 2007 survey. SA assisted in HTLV-1 testing and interpretation of the results. TV coordinated the fieldwork for the 1997 and 2007 survey and was responsible for data entry. RSN supervised the laboratory testing and interpretation of the results for 1997 and 2007 surveys. HW was involved in the planning of the three surveys. HW and PA were involved in supervision of the surveys and interpretation of the data.

## Supplementary Material

Additional file 1 Univariate analysis of factors associated with HTLV-1, by sex in Caió, Guinea-Bissau, in 1997 (a) and 2007 (b) Click here for file
