# Supplementary material for: HTLV-1 in rural Guinea-Bissau: prevalence, incidence and a continued association with HIV between 1990 and 2007
Source: Retrovirology. 2010 Jun 4;7:50. doi: 10.1186/1742-4690-7-50 (PMC2894744; doi:10.1186/1742-4690-7-50)
Supplement: Additional file 1 — Univariate analysis of factors associated with HTLV-1, by sex in Caió, Guinea-Bissau, in 1997 (a) and 2007 (b) [file 1742-4690-7-50-S1.PDF]

Table 2a. - Univariate analysis of factors associated with HTLV-1, by sex in Caió, Guinea-Bissau in 1997

	MEN			WOMEN		
	HTLV-1 cases/total (%)	OR (95% CI) Crude	P value	HTLV-1 cases/total (%)	OR (95% CI) Crude	P value
**Total**	41/1,148 (3.6)			133/1,819 (7.3)		
**Age group**						
15-24	13/481 (2.7)	1	0.004	13/512 (2.5)	1	<0.001
25-34	3/224 (1.3)	0.5 (0.1-1.7)		15/410 (3.7)	1.5 (0.7-3.1)	
35-44	2/109 (1.8)	0.7 (0.2-3.0)		13/221 (5.9)	2.4 (1.1-5.3)	
45-54	6/100 (6.0)	2.3 (0.8-6.2)		26/210 (12.4)	5.4 (2.7-10.8)	
55-64	8/74 (10.8)	4.4 (1.7-10.9)		25/198 (12.6)	5.6 (2.8-11.1)	
65-max	9/160 (5.6)	2.2 (0.9-5.1)		41/268 (15.3)	7.1 (3.8-13.6)	
Younger (15-44)	18/814 (2.2)	1	<0.001	41/1,143 (3.6)	1	<0.001
Older (45-max)	23/334 (6.9)	3.3 (1.7-6.1)		92/676 (13.6)	4.3 (2.9-6.3)	
**HIV status**[a]						
HIV-negative	32/1,057 (3.0)	1	0.004	85/1,635 (5.2)	1	<0.001
HIV-positive	9/91 (9.9)	3.5 (1.6-7.6)		48/184 (26.1)	6.4 (4.3-9.4)	
**HIV-1**						
Negative	39/1,129 (3.5)	1	0.2	126/1,792 (7.0)	1	0.003
Positive	2/19 (10.5)	3.3 (0.7-14.7)		7/27 (25.9)	4.6 (1.9-11.1)	
**HIV-2**						

Negative	35/1,081 (3.2)	1	0.04	100/1,689 (5.9)	1	<0.001
Positive	6/67 (9.0)	2.9 (1.2-7.3)		33/130 (25.4)	5.3 (3.4-8.3)	
**HIV-dual**						
Negative	40/1,143 (3.5)	1	0.2	125/1,792 (7.0)	1	<0.001
Positive	1/5 (20.0)	6.9 (0.7-63.1)		8/27 (29.6)	5.6 (2.4-13.0)	
**Marital status**						
Single	14/612 (2.3)	1	0.06	9/304 (3.0)	1	<0.001
Married	23/471 (4.7)	2.1 (1.1-4.1)		66/1,202 (5.5)	1.9 (0.9-3.9)	
Widowed	3/32 (9.4)	4.4 (1.2-16.2)		48/262 (18.3)	7.5 (3.6-15.7)	
Divorced	2/31 (6.1)	2.8 (0.6-12.7)		10/51 (19.6)	8.0 (3.1-20.8)	
**Number of children (alive or dead)**[b]						
0				7/262 (2.7)	1	<0.001
1-2				20/520 (5.1)	1.7 (0.7-3.9)	
3-4				23/574 (6.7)	2.3 (1.0-5.2)	
5 or more				83/460 (10.2)	3.6 (1.7-7.5)	
**Number of children that died**						
Never had children				7/262 (2.7)	1	<0.001
0				21/520 (4.0)	1.3 (0.6-3.1)	
1-2				51/574 (8.9)	3.1 (1.5-6.6)	
3 or more				54/463 (11.7)	4.2 (2.0-9.0)	
**Area of living**						
Peripheral area	11/393 (2.8)	1	0.3	22/616 (3.6)	1	<0.001
Central area	30/755 (4.0)	1.4 (0.7-2.9)		111/1,203 (9.2)	2.6 (1.7-4.2)	

**TPHA**[c]						
Negative	27/920 (2.9)	1	0.03	87/1,440 (6.0)	1	<0.001
Positive	14/228 (6.1)	2.2 (1.1-4.2)		46/376 (12.2)	2.1 (1.5-3.1)	
**Ever used a condom**[d]						
No	30/775 (3.9)	1	0.4	126/1,631 (7.7)	1	0.4
Yes	6/222 (2.7)	0.7 (0.3-1.7)		5/90 (5.6)	0.7 (0.3-1.8)	
**How often condom use in last month**[e]						
None	14/927 (4.4)	1	0.2	131/1,696 (8.3)	1	-
At least once	1/69 (1.5)	0.3 (0.0-2.3)		0/25 (-)	-	
**Ever received blood transfusion**[f]						
No	41/1,133 (3.6)	1	-	127/1,759 (7.2)	1	0.4
Yes	0/15 (-)	-		6/60 (10.0)	1.4 (0.6-3.4)	

OR Odds Ratio; CI Confidence Interval

[a] HIV positive includes HIV-1, HIV-2 and HIV-dual infections.

[b] 3 missing data

[c] TPHA: Treponema Pallidum Haemagglutination Test (Microsyph-TP; Porton Cambridge, Newmarket, UK); 3 missing data for women

[d] 151 missing data for men; 98 missing data for women

[e] 152 missing data for men; 98 missing data for women

[f] this information was not obtained in the 2007 survey but was included here because of its association found in Bissau (ref Larsen JAIDS 2000, da Silva AIDS 2009)

Table 2b. - Univariate analysis of factors associated with HTLV-1, by sex in Caió, Guinea-Bissau in 2007

	MEN			WOMEN		
	HTLV-1 cases/total (%)	OR (95% CI) Crude	P value	HTLV-1 cases/total (%)	OR (95% CI) Crude	P value
**Total**	36/1,158 (3.1)			96/1,734 (5.5)		
**Age group**						
15-24	10/451 (2.2)	1	0.003	18/515 (3.5)	1	<0.001
25-34	11/289 (3.8)	1.7 (0.7-4.2)		6/369 (1.6)	0.5 (0.2-1.2)	
35-44	2/180 (1.1)	0.5 (0.1-2.3)		13/303 (4.3)	1.2 (0.6-2.6)	
45-54	2/85 (2.4)	1.1 (0.2-4.9)		9/152 (5.9)	1.7 (0.8-4.0)	
55-64	1/66 (1.5)	0.7 (0.1-5.4)		24/166 (14.5)	4.7 (2.5-8.8)	
65-max	10/87 (11.5)	5.7 (2.3-14.2)		26/229 (11.4)	3.5 (1.9-6.6)	
Younger (15-44)	23/920 (2.5)	1	0.03	37/1,187 (3.1)	1	<0.001
Older (45-max)	13/238 (5.5)	2.2 (1.1-4.5)		59/547 (10.8)	3.8 (2.5-5.7)	
**HIV status**[a]						
HIV-negative	32/1,095 (2.9)	1	0.2	74/1,577 (4.7)	1	<0.001
HIV-positive	4/63 (6.4)	2.3 (0.8-6.6)		22/157 (14.0)	3.3 (2.0-5.5)	
**HIV-1**						
Negative	35/1,122 (3.1)	1	1.0	90/1,665 (5.4)	1	0.5

Positive	1/33 (3.0)	1.0 (0.1-7.3)		4/52 (7.7)	1.5 (0.5-4.1)	
**HIV-2**						
Negative	33/1,128 (2.9)	1	0.06	78/1,629 (4.8)	1	<0.001
Positive	3/27 (11.1)	4.2 (1.2-14.5)		16/88 (18.2)	4.4 (2.5-8.0)	
**HIV-dual**						
Negative	36/1,155 (3.1)	-	-	94/1,717 (5.5)	1	0.3
Positive	0/3 (-)	-		2/17 (11.8)	2.3 (0.5-10.2)	
**Marital status**						
Single	15/644 (2.3)	1	0.006	9/433 (2.1)	1	<0.001
Married	17/478 (3.6)	1.5 (0.8-3.1)		46/913 (5.0)	2.5 (1.2-5.2)	
Widowed	4/21 (19.1)	9.9 (3.0-32.9)		37/342 (10.8)	5.7 (2.7-12.0)	
Divorced	0/15 (-)	-		4/46 (8.7)	4.5 (1.3-15.2)	
**Number of children (alive or dead)[b]**						
0				7/305 (2.3)	1	0.001
1-2				13/350 (3.7)	1.6 (0.6-4.2)	
3-4				19/335 (5.7)	2.6 (1.1-6.2)	
5 or more				55/720 (7.6)	3.5 (1.6-7.8)	
**Number of children that died[c]**						
Never had children				7/305 (2.3)	1	<0.001
0				22/543 (4.1)	1.8 (0.8-4.3)	
1-2				26/493 (5.3)	2.4 (1.0-5.5)	

3 or more				39/369 (10.6)	5.0 (2.2-11.4)	
**Area of living**						
Central area	10/351 (2.9)	1	0.7	29/583 (5.0)	1	0.5
Peripheral area	26/807 (3.2)	1.1 (0.5-2.4)		67/1,151 (5.8)	1.2 (0.8-1.8)	
**TPHA**[d]						
Negative	25/1,033 (2.4)	1	0.001	78/1,481 (5.3)	1	0.3
Positive	11/125 (8.8)	3.9 (1.9-8.1)		18/253 (7.1)	1.4 (0.8-2.3)	
**Ever used a condom**						
No	19/529 (3.6)	1	0.4	77/1,278 (6.0)	1	0.1
Yes	17/629 (2.7)	0.7 (0.4-1.4)		19/456 (4.2)	0.7 (0.4-1.1)	
**How often condom use in last month**[e]						
None	8/296 (2.7)	1	0.6	10/286 (3.5)	1	0.08
At least once	28/862 (3.3)	1.2 (0.5-2.7)		86/1,448 (5.9)	1.7 (0.9-3.4)	

OR Odds Ratio; CI Confidence Interval

[a] HIV positive includes HIV-1, HIV-2 and HIV-dual infections.

[b] 24 missing data

[c] 24 missing data

[d] TPHA: Treponema Pallidum Haemagglutination Test (Microsyph-TP; Porton Cambridge, Newmarket, UK); 6 missing data for men; 4 missing data for women

[e] 18 missing data for women
